# Over 300 km Dispersion of Wild Boar during Hot Summer, from Central Poland to Ukraine

**DOI:** 10.3390/ani14010170

**Published:** 2024-01-04

**Authors:** Bartłomiej Popczyk, Daniel Klich, Paweł Nasiadka, Angelika Nieszała, Krzysztof Gadkowski, Maria Sobczuk, Marek Balcerak, Piotr Kociuba, Wanda Olech, Ludwik Purski

**Affiliations:** 1 Department of Genetics and Animal Conservation, Warsaw University of Life Sciences, Ciszewskiego 8, 02-786 Warsaw, Polandpawel_nasiadka@sggw.edu.pl (P.N.); angelika_nieszala@sggw.edu.pl (A.N.); maria_sobczuk@sggw.edu.pl (M.S.);; 2 GIGACO Ltd., Świeradowska 47, 02-662 Warsaw, Poland; krzysztof@gadkowski.pl (K.G.);; 3 Department of Animal Breeding, Warsaw University of Life Sciences, Ciszewskiego 8, 02-786 Warsaw, Poland; marek_balcerak@sggw.edu.pl; 4 Institute of Mathematics, Informatics and Landscape Architecture, The John Paul II Catholic University of Lublin, Konstantynów 1H, 20-708 Lublin, Poland; piotr.kociuba@kul.pl

**Keywords:** movement, *Sus scrofa*, distance, temperature, precipitation

## Abstract

**Simple Summary:**

A wild boar usually covers short distances, but sometimes it ventures far from its usual habitat. The objective of this study was to document a rare instance of an extended wild boar migration from Poland to Ukraine. We investigated the extent to which the wild boar’s movement correlated with air temperature and rainfall, comparing it with a wild boar that did not undertake such lengthy migrations. The findings revealed that migrating wild boars tended to cover greater distances during warmer days. This outcome, unexpectedly, may be attributed to the wild boar’s presence in an unfamiliar environment.

**Abstract:**

The movement of wild boars is a complex process influenced by both internal conditions and external factors. Despite their typically sedentary lifestyle, dispersion constitutes an integral element of the wild boar’s behavior. This report documents the longest observed wild boar dispersal, involving a collared two-year-old male near Warsaw, Poland. The aim of this study was to present the characteristics of movement during the “nomadic phase”, drawing comparisons with the “sedentary phase”. The other aim was to evaluate the influence of meteorological factors on the minimum daily travel distance of the wild boar. We collected data from two-year-old males. The first exhibited long-distance dispersal and the second only demonstrated local movements. We calculated the minimum daily distance of both wild boars based on collar locations and calculated basic statistics of movement. We used a generalized linear model with a gamma distribution and log link function to assess the potential impact of weather conditions on the minimum daily distance of wild boars. We tested maximum daily temperature, average daily temperature, and the sum of daily precipitation. The wild boar during a “nomadic phase” covered a total of 922 km with a mean minimum daily movement of 6 km. The dispersion distance was 307 km. The highest value of the minimum daily distance reached 31.8 km/day. The second wild boar (near Warsaw) covered a mean minimum daily distance of 1.4 km; the highest value of the minimum daily distance was 3.9 km. Both wild boars exhibited no dependence of minimum daily distance on weather conditions. However, when intensive and non-intensive dispersion were analyzed separately, it was demonstrated that the maximum daily temperature positively influenced the minimum daily distance. We speculate that the wild boar was forced to search for water sources after dark on hot days, which induced a longer traveling distance in an unfamiliar environment. This study highlights the significant spatial capabilities of wild boar in the transmission of genes or pathogens. We speculate that extended daily distances during the initial “nomadic phase” might suggest a panicked escape from a perceived threat. It is plausible that the wild boar found improved shelter within tall cereal crops in July and August, which resulted in lower daily distances.

## 1. Introduction

Animal movement plays a pivotal role in ecology, providing valuable insights into spatial behavior [1,2]. Movement is intricately linked with home range size [3], encompassing habitat selection across various spatial and temporal scales [3,4]. Contemporary studies on animal movement often employ novel techniques and analytical tools, e.g., [5,6,7]. These investigations also extend to the wild boar, a species demonstrating a remarkable ability to colonize and adapt to diverse habitats including those altered by human activities [8].

The movement of wild boars is a complex process influenced by both internal conditions and external factors [9]. Despite their typically sedentary lifestyle, dispersion constitutes an integral element of the wild boar’s “nomadic phase” distinguished from the “sedentary phase” during which the animal returns to the same resting site [10,11]. A crucial factor leading to increased travel distances in wild boars is disturbance, particularly as a consequence of hunting. Hunting can result in an expanded range and altered areas utilized by wild boars, indicative of dispersion [10,12]. However, dispersion is also a natural phenomenon associated with individual growth as yearlings leave their natal areas [13,14]. Wild boar movement typically occurs over short distances, ranging from a few kilometers to sometimes several dozen kilometers [15,16,17,18,19]. Occasionally, however, much longer distances have been observed, with the farthest recorded dispersal using telemetry collars reaching approximately 100 km from the release site [20]. There are also documented cases of marked wild boars dispersing distances exceeding 250 km [21].

Effects of weather conditions have been found in ungulate species, for example, a negative effect of temperature and a positive effect of precipitation have been observed on white-tailed deer movement [22]. Similarly, a negative effect of high temperatures has been observed on the movement of the mule deer [23], but high temperatures increased the odds of moose traveling in bogs and mixed forests [24]. For wild boar, the effect of precipitation has been confirmed, but the impact of temperature on movement has been studied mainly in relation to the season (cold or hot season) [25,26,27,28,29].

This report documents the longest observed wild boar dispersal involving a collared two-year-old male near Warsaw, Poland. The journey commenced in May 2021 in Warsaw and extended to Ukraine by August 2021. The primary objective of this study was to present the characteristics of movement during the “nomadic phase” and to draw comparisons with the “sedentary phase”. Additionally, the study aimed to evaluate the influence of meteorological factors, specifically temperature and precipitation, on the daily travel distance of the wild boar.

## 2. Methods

**Figure 1:**
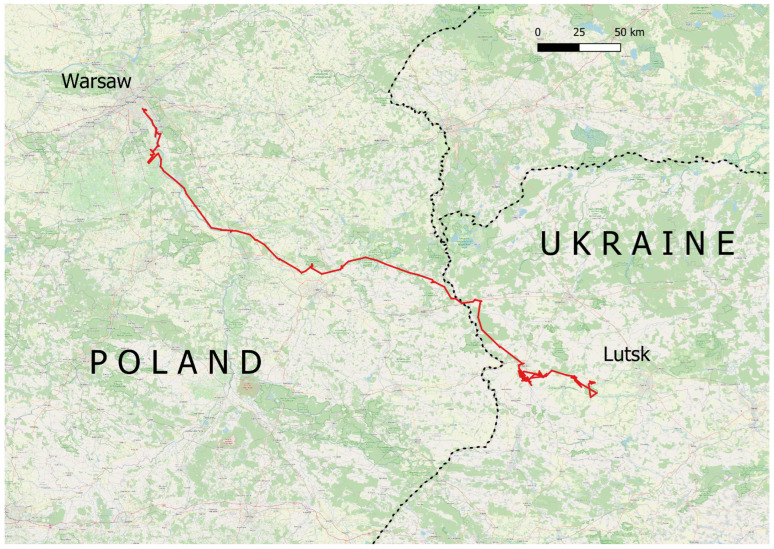
Route of wild boar GIGA 13’s journey to Ukraine (period between 14 May and 24 August 2021).

The study encompasses collared wild boars as part of a scientific project funded by the National Center for Research and Development in Poland [30]. We collected data from two-year-old males, namely GIGA 13, exhibiting long-distance dispersal, and GIGA 17, only demonstrating local movements, among other collared wild boars. Both individuals were captured in hunting district no. 456 (Piaseczno district) and equipped with GPS/GSM transmitters in 2020. We obtained all the necessary permits as detailed in the study by Popczyk et al. [30]. The collars remained operational in 2021, coinciding with the occurrence of the long-distance dispersal. The collars were originally configured to transmit one location measurement every 6 h during the study period. However, technical issues, such as intermittent module operation and the lack of GPS signal, led to a reduced number of measurements on certain days. Upon crossing the Polish–Ukrainian border, roaming was activated to ensure continued data retrieval. The data analysis focused on the spring and summer period from 1 March 2021 to 24 August 2021, the point when the animal’s collar ceased operation in Ukraine (Figure 1).

**Figure 2:**
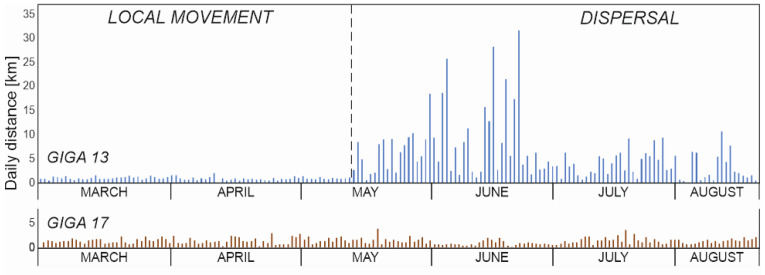
Daily movement minimum distance of wild boars GIGA 13 (traveling to Ukraine) and GIGA 17 (utilizing local habitats in the spring and summer of 2021).

We used the LSD method (straight-line distance between location points), which is commonly used in daily distance assessment, and represented a minimum daily distance, e.g., [27,31,32]. Using QGIS 3.30 software and a Python script developed through the PyQGIS library, we calculated the daily distance of both wild boars based on GPS locations from collars. Each iteration of the script analyzed two locations—the previous one and the current one—calculating the distance in meters considering the WGS84 ellipsoid. The minimum daily distance was calculated only for days for which there were at least two GPS locations and measurements from the previous or next day. We saved results in the attribute table of the layer and exported them to an .xlsx file. We divided the analyzed the period for GIGA 13 into the “sedentary phase” and “nomadic phase”. The “nomadic phase” commenced on 14 May and was marked by a sudden increase in minimum daily distance to over 2.7 km followed by even greater increases in the subsequent days (Figure 2). The end of the “nomadic phase” coincided with the termination of the collar’s operation as long minimum daily distances persisted thereafter. For both periods (local movement and dispersion), we calculated the maximum values of minimum daily distance, the average value, the standard deviation, and the median. These statistics were also computed for the entire analyzed period for the GIGA 17 male.

Due to the fact that the effect of weather on wild boar’s daily distance has not been studied so far, we made an attempt to investigate this phenomenon with significant daily fluctuations of movement. To evaluate the influence of weather conditions on wild boar movement, we gathered temperature values (maximum, minimum, and average daily temperature) and total rainfall for each day. We obtained data from the nearest weather stations to the respective wild boar locations, with daily values accessible on the Institute of Meteorology and Water Management’s website (https://danepubliczne.imgw.pl, accessed on 4 October 2023). Before conducting statistical analysis, we assessed the cross-correlation of temperature data including daily maximum, daily minimum, and daily average values. A moderately strong correlation was observed between maximum and minimum values (Pearson’s r = 0.64); therefore, minimum temperatures were excluded from the analysis. Additionally, the normal distribution of the dependent variable, i.e., the minimum daily distance, was examined using the Shapiro–Wilk test, revealing a lack of normal distribution.

Subsequently, we used a generalized linear model with a gamma distribution and log link function to assess the potential impact of weather conditions on the minimum daily distance of wild boars. The weather variables considered were maximum daily temperature (°C), average daily temperature (°C), and the sum of daily precipitation (mm). Model selection was conducted based on the Akaike information criterion (AIC) [33]. All possible combinations of explanatory variables were explored and compared according to a null intercept-only model. The best-fit models, identified through the lowest AIC score, were chosen, with preference given to simpler models among those with a ΔAIC < 2. The analysis covered both animals for the period from 14 May to 24 August 2021. We developed two additional models for the GIGA 13 male distinguishing between the periods of intense dispersal (14 May–23 June 2021) and non-intense dispersal (24 June–24 August 2021). In total, we used data from 96 days regarding GIGA 13 for statistical analysis (56 days for intense dispersal and 40 days for non-intense dispersal) and 102 days for wild board GIGA 17. All statistical analyses were conducted using SPSS software (version 29.0, IBM Corporation, Armonk, NY, USA).

## 3. Results

GIGA 13 covered a total minimum distance of 998 km between 1 March and 24 August 2021. During the “sedentary phase”, the wild boar traveled 76 km, with a mean minimum daily movement of 1 km (SE = 0.3 km, median = 1 km). The highest value of minimum daily movement during this period was 2.1 km/day. Starting from 14 May, daily minimum distances significantly increased; by 22 June, GIGA 13 had covered a total of 922 km, with a mean minimum daily movement of 6 km (SE = 5.9 km, median = 4.5 km). The dispersion distance was 307 km. The highest minimum daily distance reached 31.8 km/day. After 23 June, the wild boar no longer traveled extremely long distances, with only one instance where its daily minimum distance exceeded 10 km (Figure 2). During the entire period, the GIGA 17 male traveled a minimum distance of 238 km, with a mean minimum daily distance of 1.4 km (SE = 0.6 km, median = 1.3 km) and the highest value of the minimum daily distance being 3.9 km. Daily distances showed no visible differences between months (Figure 2).

**Figure 3:**
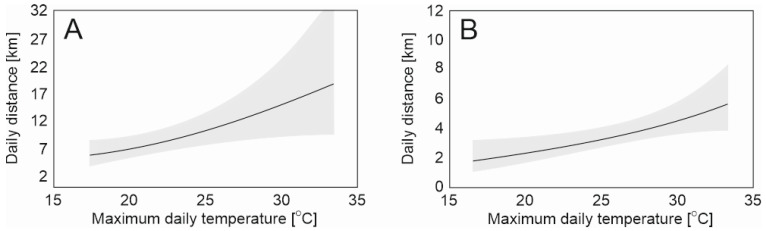
Effect of maximum daily temperature on the minimum daily distance of wild boar GIGA 13 during (**A**) intensive (between 14 May and 23 June) and (**B**) non-intensive (between 24 June and 24 August) dispersal. The graph presents the expected value of minimum daily distance (solid line) and 95% confidence intervals (shaded area).

Both wild boars exhibited no dependence of daily distance on weather conditions throughout the entire dispersal period of GIGA 13. In both cases, the null model showed the lowest AIC values. However, in the divided period variant for the GIGA 13 male, the influence of the maximum daily temperature on the minimum daily distance was demonstrated during both the period of intensive dispersion (Figure 3A) and the period of non-intensive dispersion (Figure 3B). During the period of intensive dispersal, the increase in daily movement was more pronounced (B = 0.073, SE = 0.295, *p* = 0.013) than during non-intensive dispersal (B = 0.067, SE = 0.244, *p* = 0.006). Nevertheless, lower error was observed in the period of non-intensive dispersion (Figure 3). Other explanatory variables (average temperature and precipitation) could not explain the minimum daily distance and were excluded as a result of model selection.

## 4. Discussion

Dispersal in wild boars is commonly observed in juveniles, although instances also occur in adults [13,21]. Notably, the longest recorded dispersal on a collared wild boar (100 km) was documented for an adult female [20]. In our study, we observed an unprecedented dispersal distance performed by a two-year-old male wild boar, surpassing not only the collared adult female but also individuals observed through the mark–recapture method [20,21]. GIGA 13 also achieved the longest recorded daily distances, even exceeding 30 km/day. These distances surpassed those covered by wild boars dispersing as a result of collective hunting, a factor known to significantly influence the spatial activity of wild boars [12,20,28].

While the number of daily GPS locations in our study may be considered limited, it is indicative of the covered route likely being much longer. Despite the small number of daily locations, the dynamics of wild boar movements on specific days, characterized by both long and short minimum daily distances, were captured. This pattern was similar to findings by Jerina et al. [20]. However, a novel observation in our study was the change in the dynamics of wild boar movement. Following a period of intensive dispersion, the distances covered diminished approximately a month later. These were not repetitive, short migrations indicative of the “sedentary phase”, but rather suggested a search for a settling place as the minimum distances covered remained considerably longer than in the pre-dispersion period and compared to that covered by the GIGA 17 male. During this stage, several days of short minimum daily distances characteristic of the “sedentary phase” were observed followed immediately by a several-fold, even ten-fold, increase in minimum daily distance (Figure 2). Interestingly, this stage persisted longer than the intensive dispersion phase.

Our study has also demonstrated that the maximum temperature influences the minimum daily travel distance of wild boar. However, this effect became evident only after dividing the dispersion stage into intensive and non-intensive phases. Although the influence of rain on wild boar movement has not been observed in previous studies, the influence of snow cover and snowfall has been confirmed [25,27]. Nevertheless, the most critical factor was ambient temperature. Distances covered and home ranges decrease during colder seasons [26,28]. According to Dexter [34], temperature affects the habitat selection of feral pigs towards shady areas. In winter, a direct negative impact of low temperatures on wild boar movements has also been confirmed [27]. Campbell and Long [35] demonstrated a positive effect of temperature on daily wild boar movement only during spring, with no such effect noted during summer. The results of our study shed new light on wild boar movement and do not necessarily contradict previous studies. Feral pigs were active during the daylight, so temperature compelled them to seek shaded habitats. Wild boars, including GIGA 13, are nocturnally active and high temperatures probably do not directly influence their behavior. We speculate that on a hot day, likely combined with a lack of rainfall and high evaporation, the wild boar was forced to search for water sources after dark. Perhaps this exploration in an unfamiliar environment induced a longer distance traveled during the night.

## 5. Conclusions

The presented study provides new insights into the wild boar movement pattern, although it is based solely on a single individual. Contrary to the typical sedentary lifestyle of wild boars, the GIGA 13 individual attempted extensive territory penetration, as demonstrated through its route. Despite encountering challenges such as hunters and potential collisions with vehicles, the wild boar managed to cover such a vast distance. During the dispersion, the boar crossed the Vistula River twice near Warsaw and crossed the highway through an underpass along a small watercourse. These barriers did not seem to have deterred the boar. This highlights the significant spatial capabilities of this species in the transmission of genes or pathogens. Extended daily distances during the initial dispersion phase might suggest a panicked escape from a perceived threat. Subsequently, the movement dynamics diminished and the cause remains unknown. It is plausible that the wild boar found improved shelter within the tall cereal crops in July and August, which also offer abundant food resources. A definitive interpretation of the observed phenomenon is elusive, underscoring the need for additional telemetry studies involving a larger number of animals. Such studies would contribute to a more comprehensive understanding of this facet of wild boar ecology in a dynamically changing agricultural landscape. Furthermore, they could potentially lead to the development of more effective methods for managing the population of this species.

## Data Availability

The data presented in this study are available on request from the corresponding author.
